# Complete Genome Sequence of a CTX-M-15-Producing *Klebsiella pneumoniae* Outbreak Strain from Multilocus Sequence Type 514

**DOI:** 10.1128/genomeA.00742-15

**Published:** 2015-07-09

**Authors:** Laura Becker, Boyke Bunk, Christoph Eller, Matthias Steglich, Yvonne Pfeifer, Guido Werner, Ulrich Nübel

**Affiliations:** a Robert Koch Institute, FG13 Division of Nosocomial Pathogens and Antibiotic Resistances, Department of Infectious Diseases, Wernigerode Branch, Wernigerode, Germany; b Leibniz Institute DSMZ, Braunschweig, Germany; c German Centre for Infection Research (DZIF), Partner Site Hannover-Braunschweig, Braunschweig, Germany

## Abstract

We report here the genome sequence of a multidrug-resistant *Klebsiella pneumoniae* strain, which caused an outbreak in a neonatal ward in 2011. The genome consists of a single chromosome (5,278 kb) and three plasmids (362 kb, 5 kb, and 4 kb).

## GENOME ANNOUNCEMENT

*Klebsiella pneumoniae* is a nosocomial pathogen that poses a particular threat to neonates and immunocompromised patients (1). Here, we announce the complete genome sequence of an extended-spectrum β-lactamase (ESBL)-producing *K. pneumoniae* strain from multilocus sequence type 514 (ST514). This strain (isolate 234-12) was isolated from a blood culture in 2011 during an outbreak on a neonatal intensive care unit in Germany (2).

Whole-genome sequencing was performed on a PacBio RSII system (Pacific Biosciences, USA) by a commercial service provider (GATC Biotech, Konstanz, Germany). In addition, genomic DNA was sequenced by applying the Nextera XT library kit and a MiSeq v3 reagent kit with 600 cycles on a MiSeq sequencer (Illumina, USA). A total of 53,705 PacBio reads with a mean read length of 6,143 bp were assembled using the RS_HGAP_Assembly.3 protocol implemented in SMRT Portal version 2.3.0. Chromosomal contigs were scaffolded, and the remaining gaps were closed on the basis of bridging PacBio reads, resulting in a closed circular chromosomal sequence of 5,278 kb. Two additional contigs exhibited overlapping ends and thus were circularized to plasmid sequences with 5 kb and 362 kb, respectively. Gel electrophoresis confirmed the sizes of these two plasmids and revealed the presence of a third plasmid with a size of 4 kb. The sequence of the 4-kb plasmid had not been assembled from PacBio data initially, but it was retrieved by assembling Illumina reads *de novo* using Geneious version 7.1.4 (3). Illumina reads were mapped onto all replicons to improve the sequence quality to QV60. Finally, all four replicons were structurally confirmed by mapping the PacBio reads (RS BridgeMapper.3 protocol). Annotation was performed using Prokka 1.8 (4) and manually supplemented where appropriate. PacBio sequencing revealed three sequence motifs with methylated adenine residues (N^6^-methyladenine, 6 mA): GATC, CCAYN_5_TCC, and GGAN_5_RTGG.

The chromosome carries three resistance genes, and the 362-kb plasmid carries 13 resistance genes, including *bla*_CTX-M-15_ (Kpn23412_5431), which was responsible for the ESBL phenotype of the bacterium (5). Antimicrobial susceptibility testing confirmed resistance to β-lactams (including penicillin and third-generation cephalosporins), fluoroquinolones, aminoglycosides, chloramphenicol, tetracycline, and sulfamethoxazole-trimethoprim, respectively. Furthermore, the 362-kb plasmid exhibits four replication-related *rep* genes, including *repC* (Kpn23412_5167), which is identical to the *repC* in the reference sequence for replicon type IncQ1 (6), and *repA* (Kpn23412_5166). Further, *repB1* (Kpn23412_5315) suggested replicon type IncFIB (Mar), and *repB2* (Kpn23412_5428) suggested IncHI1B. The presence of multiple replicons on individual plasmids may broaden their host range and avoid incompatibility by switching between alternative replication mechanisms (7). In contrast, the replication-related RNA1 (Kpn23412_5489 and Kpn23412_5493) was identified on both small plasmids, but no previously classified replicon types could be assigned.

### Nucleotide sequence accession numbers. 

The genome sequences of *K. pneumoniae* isolate 234-12 were deposited at NCBI GenBank and assigned accession numbers CP011313 to CP011316.


